# Bypassing the Gut–Lung Axis *via* Microbial Metabolites: Implications for Chronic Respiratory Diseases

**DOI:** 10.3389/fmicb.2022.857418

**Published:** 2022-05-03

**Authors:** Edyta Bulanda, Tomasz P. Wypych

**Affiliations:** Laboratory of Host-Microbiota Interactions, Nencki Institute of Experimental Biology, Polish Academy of Sciences, Warsaw, Poland

**Keywords:** microbiome, probiotics, metabolites, postbiotics, lung immunity, lung disease

## Abstract

The gut microbiome engages in constant interactions with the immune system, laying down the fundamentals of what we perceive as health or disease. The gut microbiota acts locally in the intestines and distally in other organs, such as the lungs. This influence (termed “the gut–lung axis”) constitutes the basis for harnessing the microbiome to prevent or treat chronic respiratory diseases. Within this context, two approaches gained the most attention: the diet interventions (which shape the microbiome) and the probiotics (which exert beneficial effects directly on the host). Microbial products, which constitute a means of communication along the gut–lung axis, are only now emerging as a new class of potential therapeutics. Here, we provide a comprehensive overview of microbial products active in the airways, describe the immunological mechanisms they trigger, and discuss their clinical advantages and pitfalls.

## Introduction

### The Lung Microbiota

Lungs are not sterile. Instead, they harbor a detectable microbial community that influences respiratory homeostasis. In health, this “lung microbiome” is dynamic and fluctuating, as it is constantly being shaped by three perpetuating forces: (i) microbial immigration, (ii) microbial elimination, and (iii) microbial proliferation. The immigration involves direct inhalation of microorganisms, their micro-aspiration from the oral cavity/upper airways, and further dispersion along the mucosal surfaces. Elimination involves clearance mechanisms (e.g., cough and mucociliary clearance), and immune mechanisms (e.g., phagocytosis and immune exclusion by mucosal antibodies). Finally, the reproduction rate is determined by the local environment, including the inflammatory status, microbe–microbe competition, and physical properties of the airways (temperature, pH, and tensions caused by airflow). In a healthy lung, all of these forces orchestrate the maintenance of a low and diverse microbial biomass, which sets the immunological tone in the airways (Huffnagle et al., 2017). The impairment of these forces may lead to the distortion of the lung microbiome with health consequences. Indeed, observational studies in humans pointed to substantial differences between the microbiomes of healthy subjects and those suffering from respiratory disorders (Yagi et al., 2021), while animal models provided evidence of the causal relationship between the two (Gollwitzer et al., 2014; Yadava et al., 2016; Jin et al., 2019).

### The Gut–Lung Axis

In addition to the local effects of the lung microbiome, respiratory homeostasis is further shaped by the central microbial hub in the body, the gut. Bacterial products, including pathogen-associated molecular patterns (PAMPs), can be directly transferred from the gut to the lungs and modulate the immunity of the airways. Lipopolysaccharide (LPS), when injected intrarectally into mice, was found to restore the ability of antibiotic-treated mice to clear influenza virus infection (Ichinohe et al., 2011) and protect against allergic airway inflammation (Qian et al., 2018). Gut microbes can also modulate the function of immune cells that subsequently migrate into the lungs. The relevance of this cross-talk was found in the case of influenza virus infection. The host-derived lipocalin-2 (lcn-2) protected mice against influenza virus infection in a microbiome-dependent manner. Specifically, changes in the microbiome caused by lcn-2 deficiency impaired the function of CD103^+^ dendritic cells, causing aberrant CD8^+^ T cell proliferation, and contributing to immunopathology (Watzenboeck et al., 2021). The microbiome also promoted the trafficking of ILC2, and ILC3 cells along the gut–lung axis in the context of *Nippostrongylus brasiliensis* infection, and pneumonia, respectively (Gasteiger et al., 2015; Gray et al., 2017). Though beneficial in the above situations, the microbe-induced cell migration along the gut–lung axis may also be detrimental in certain situations. For example, segmented filamentous bacteria initiated the migration of autoreactive Th17 cells into the airways to promote lung pathology (Bradley et al., 2017). This example illustrates one mechanism through which host–microbe interactions in the gut can instigate lung disease. It remains unclear how commonly such events contribute to the development of pulmonary disease. However, intestinal dysbiosis in lung disease is well documented. Infants with the reduced relative abundance of *Bifidobacterium*, *Faecalibacterium*, and *Akkermansia*, and increased relative abundance of *Candida* and *Rhodotorula* in the gut, were in the highest risk group for developing asthma symptoms later in life (Fujimura et al., 2016). Also, a lower abundance of *Akkermansia municipila* was linked to higher severity of asthma in adults (Michalovich et al., 2019) and finally, intestinal bacteria belonging to the *Christensenellaceae* family, positively correlated with asthma in three-year-old children (Lee-Sarwar et al., 2019). Shifts in the microbiota composition were also evident in COPD patients and associated with decreased lung function. Although variable among individuals, key differentiators were *Streptococcus* species and multiple members belonging to the family *Lachnospiraceae* (Bowerman et al., 2020). In cystic fibrosis, the differences in the gut microbiota were also evident, albeit equally variable between studied cohorts (Zhang et al., 2020). Reduced levels of *Bacteroidetes* seemed to be a reproducible trait (Nielsen et al., 2016; Burke et al., 2017; Fouhy et al., 2017; Antosca et al., 2019), which might contribute to changes in certain metabolic pathways within the gut microbiota. For instance, Fouhy et al. demonstrated that a skewed ratio of Firmicutes/Bacteroidetes associated with enhanced lipid, protein, and xenobiotic metabolism, as well as with differences in fecal metabolite profile (Fouhy et al., 2017). However, the functional significance of these changes is yet to be evaluated.

Finally, the existence of an active dialogue between the gut and the lungs is reinforced by the pulmonary manifestations of various gastrointestinal diseases (Ji et al., 2014). For instance, inflammatory bowel disease is associated with asthma (Kuenzig et al., 2018), chronic obstructive pulmonary disease (Raftery et al., 2020), idiopathic pulmonary fibrosis (Kim et al., 2020), bronchitis (Bernstein et al., 2005), bronchiectasis (Ott and Scholmerich, 2013), and cryptogenic organizing pneumonia (Betancourt et al., 2011). In many instances, it remains elusive what condition instigates the other. Given the bidirectionality along the gut–lung axis, it is likely that both scenarios can occur. This is exemplified by the IBD–asthma association where, on one hand, children with a very early onset of IBD were at the highest risk of developing asthma later in life (Barbiellini Amidei et al., 2020), and on the other, children first diagnosed with asthma had an increased risk for developing IBD at the later age (Kuenzig et al., 2018).

## Microbial Metabolites—Messengers Along the Gut–Lung Axis

**Figure 1:**
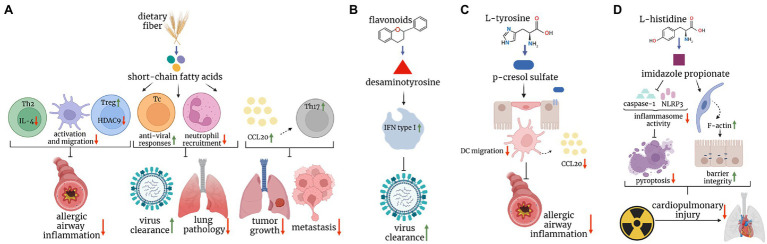
Postbiotics in mouse models of lung disease. **(A)** Short-chain fatty acids, major breakdown products of dietary fiber inhibit dendritic cell and Th2 responses, and augment regulatory T cell response, protecting mice against allergic airway inflammation. They also boost protective CD8^+^ T cell immunity against the influenza virus, and limit cytotoxic effects of neutrophils, leading to increased viral clearance and reduced immunopathology, respectively. Finally, they enhance the CCL20-Th17 axis to reduce tumor growth and metastasis. **(B)** Desaminotyrosine, derived from the metabolism of flavonoids augments type I interferon signaling in phagocytes and protects mice against influenza virus infection. **(C)** P-cresol sulfate, a product of L-tyrosine metabolism, inhibits the production of CCL20 and dendritic cell migration in the lungs, ultimately reducing allergic airway inflammation. **(D)** Imidazole propionate, derived from L-histidine, reduces the inflammasome activity and increases F-actin expression, leading to reduced pyroptosis and improved barrier integrity, respectively, and ultimately ameliorating cardiopulmonary injuries caused by radiation.

Microbial metabolites are low molecular weight compounds, produced by microbes throughout the normal cell cycle. Many of them derive from nutrients and may exert beneficial, neutral, or harmful effects on the host. Metabolites conferring health benefits are often referred to as postbiotics (Tsilingiri and Rescigno, 2013; Aguilar-Toala et al., 2021), the microbes that produce them as probiotics, while the nutrients that constitute the source of postbiotics and promote the growth of probiotics, as prebiotics. One way postbiotics achieve their beneficial effect in the context of disease is via immunomodulation (Figure 1). One of the most widely known prebiotics is dietary fiber, a component of many plant foods. Consumption of dietary fiber including fermentable pectin and non-fermentable cellulose led to an increase in the diversity of the gut microbiome (Jang et al., 2021), a feature associated with improved health (Mosca et al., 2016). Shifts in the microbiota composition impacted the metabolome of the host, with changes observed in the metabolism of short-chain fatty acids (SCFAs), linoleic acids, arachidonic acids, sphingolipids, and bile acids. This example illustrates that dietary fiber metabolism extends its effects beyond SCFAs as commonly assumed, but instead, triggers multiple changes within the host’s metabolite profile. These changes appear to have a substantial impact on health since a high fiber diet (HFD) led to reduced inflammatory response and lung damage caused by cigarette smoke-induced emphysema (Jang et al., 2021). Of note, although SCFA are usually being credited for such effects on health, other molecules might play a role. Conjugated linoleic acids are one such example. Consisting of 18 carbon atoms and two unsaturated hydrocarbons, they can form at least 28 isomers in nature (Banni, 2002). However, the main isoform that is converted from polyunsaturated fatty acids by the intestinal bacteria (e.g., *bifidobacterium* or *propionibacterium* spp.) is cis-9 trans-11-18:2, hereafter referred to as the CLA (Devillard et al., 2007). When supplemented from 8 weeks before the birch pollen season, CLA improved the overall wellbeing and sneezing frequency in allergic patients during the season. This was accompanied by the reduction of the inflammatory response, including the production of TNF-α, GM-CSF, IFN-*γ*, and IL-5 (Turpeinen et al., 2008). The mechanisms of action may rely on the sustained expression of PPAR-*γ* since CLA supplementation in mice prevented PPAR-γ transcript downregulation in the lung, and a PPAR-*γ* antagonist partially reversed a beneficial effect of CLA feeding in a mouse model of asthma (Jaudszus et al., 2008). However, as others pointed out, CLA can be further metabolized, either by the host or the microbiota, and their metabolites could display different immunomodulatory activities. For instance, 12,13-diHOME, a linoleic acid metabolite exacerbated the allergic airway inflammation in mice *via* PPAR-*γ* signaling in dendritic cells, which led to reduced numbers of regulatory T helper cells (Levan et al., 2019). Importantly, bacterial genes encoding for epoxide hydrolases (enzymes responsible for the generation of 12,13-diHOME) were found elevated in the stool of neonates on the trajectory to developing asthma. These genes were identified in common early life colonizers, such as *Enterococcus faecalis*, *Bifidobacterium bifidum*, *Streptococcus*, and *Lactobacillus* (Levan et al., 2019). A similar complexity has been observed in the case of sphingolipids, which contain a sphingoid base and amino alcohols, and can either be diet, host, or microbe-derived. On one hand, subcutaneous sphingosine-1-phophate (S1P) administration in mice led to increased expression of Th2/17 cytokines in the lungs 14 days after treatment and resulted in increased airway hyperresponsiveness (Roviezzo et al., 2010). On the other hand, either pharmacological or genetic impairment in sphingolipids biosynthesis increased bronchial hyperresponsiveness in mice (Worgall et al., 2013). A similar complexity has been observed in human studies. On one hand, plasma S1P concentrations positively correlated with the severity of bronchial hyperreactivity in allergic patients challenged with house dust mite (Kowal et al., 2019), and on the other, *de novo* sphingolipid biosynthesis was reduced in children in asthma (Ono et al., 2020). Last, bile acids (which composition appears to be shaped by the high fiber diet, too), may also shape immunity in the airways. Primary bile acids are synthesized in the liver, conjugated to taurine or glycine as salts, and stored in the gallbladder. After dietary fat intake, primary bile salts are released into the intestinal lumen where they help break down lipids. In the intestine, they are modified by the commensals to form secondary bile acids. One such secondary bile acid, ursodeoxycholic acid (UCDA), was shown beneficial in a mouse model of asthma. When administered intratracheally, UCDA reduced the numbers of eosinophils, DCs, and T cells in the bronchoalveolar lavage fluid, and inhibited the production of IL-13 upon mediastinal lymph node restimulation. UCDA acted upon farnesoid X receptor on DCs and skewed its ability to prime T cells into a Th1 rather than a pro-allergic Th2 subset (Willart et al., 2012). Collectively, it is possible that both the host and its microbiota contribute to the composition of sphingolipids, linoleic, and bile acids, and thus, shape the immunological consequences of lipid metabolism. Unraveling the complexity of these pathways, as well as their interactions, remain a challenge for future studies. Nevertheless, SCFAs are usually credited for the beneficial effects of a high fiber diet, and their effects on respiratory health appear more consistent (Lee-Sarwar et al., 2020). SCFAs are organic acids consisting of 1–6 carbon atoms in an aliphatic chain and are the main, and final, metabolic products of dietary fiber fermentation by the intestinal microbiota. Major SCFAs include acetate, propionate, and butyrate (Yan and Ajuwon, 2015). SCFAs were shown to influence inflammatory responses in various disease models, including arthritis, colitis, and allergic asthma (Wypych et al., 2019). Regarding the latter, one indication that SCFAs might play a role in shaping susceptibility to asthma came with the observation that vancomycin treatment in mice exacerbated ovalbumin-induced allergic airway inflammation (Russell et al., 2012). Because vancomycin depleted SCFA-producing bacteria, the authors wondered if supplementation of drinking water with a cocktail consisting of butyrate, acetate, and propionate, could dampen the allergic airway inflammation. Indeed, SCFAs supplementation ameliorated the allergic reaction *via* two major mechanisms. First, it acted directly on CD4^+^ T cells and inhibited the production of IL-4, a canonical Th2 cytokine. Second, it induced an anti-inflammatory gene expression profile in dendritic cells, resulting in attenuated DC activation, migration, and stimulation of Th2 lymphocytes. Using a reductionist approach, the authors recapitulated the effect using butyrate alone (Cait et al., 2018). However, by employing a similar strategy, Trompette et al. showed that a key metabolite within the SCFA pool to inhibit the allergic airway inflammation is propionate. Treating mice with propionate led to a gradual resolution of the allergic airway inflammation. Mechanistically, propionate enhanced, rather than impaired, the differentiation of dendritic cells. However, these DCs expressed reduced levels of MHCII and CD40, explaining their inability to propagate Th2 responses (Trompette et al., 2014). Finally, in yet another attempt to delineate the key component of SCFAs in protection against asthma, a role for acetate was noted. Thorburn et al., demonstrated that administration of acetate *in vivo* increased the frequency and suppressive activity of regulatory T cells. This effect was achieved *via* inhibition of histone deacetylase inhibitor 9 (HDAC9), which led to increased acetylation at the Foxp3 promoter and drove its enhanced expression. Strikingly, this trait was heritable and maintained in the pups of mice born to acetate-treated mice. When maintained on the acetate regimen till day 12 of life, the offspring was protected against allergic airway inflammation in adulthood, indicating that there is an epigenetic predisposition to asthma and that it depends on the mother’s diet during pregnancy and in early life (Thorburn et al., 2015). Although anti-inflammatory properties of SCFAs might suggest their negative impact in the context of infection, the data so far show otherwise. Butyrate treatment improved the survival of influenza virus-infected mice as it reduced neutrophilia and immunopathology in the airways. Butyrate acted *via* free fatty receptor 3 (FFAR3) in the bone marrow to induce migration of Ly6C^−^ patrolling monocytes into the lung tissue and their differentiation into M2 macrophages, a subset of macrophages that produced neutrophil chemoattractant, CXCL1 at a reduced rate (Trompette et al., 2018). In some cases, SCFA’s influence on the severity of inflammation might depend on its concentration/dosage. For example, Ghorbani et al. showed that treating cell lines derived from cystic fibrosis patients with a low concentration of butyrate or propionate (0.5–2.5 mM) led to augmentation of iNOS expression; however, high concertations (25–50 mM) caused the reverse effect. In addition to their effect on immune regulation, SCFAs might also directly affect pathogen growth, as exemplified by *Pseudomonas aeruginosa*, which accelerated its division at low concentrations of propionate but slowed it down at high concentrations (Ghorbani et al., 2015). The above examples suggest that metabolite dosage might be key in determining the functional outcome of the treatment. In support of this, Tian et al. showed that propionate doses dictated its immunomodulatory nature. At low levels, propionate augmented the production of IL-6 in endothelial cells and alveolar macrophages and propagated immune response during *Staphylococcus aureus*-induced pneumonia. At high levels, however, it suppressed immunity, resulting in impaired pathogen clearance (Tian et al., 2019). The dark side behind the SCFA effect on health has also been demonstrated in chronic rhinosinusitis. Blockage of the sinus mouth by large amounts of mucus led to the outgrowth of anaerobes that used mucin as a source of nutrients. As a consequence of mucin fermentation, large amounts of SCFAs were released fueling the growth of *Pseudomonas aeruginosa* and the development of chronic inflammation (Cho et al., 2020).

In addition to allergies and infections, the role of SCFAs has also been described in the context of cancer. Propionate and butyrate inhibited lung metastasis of melanoma cells. Mice fed with VSL#3, a probiotic mixture inducing SCFA production in the intestine had fewer tumor foci in the lungs due to elevated expression of C-C motif chemokine ligand 20 (CCL20) in the airways and enhanced infiltration of Th17 cells (Chen et al., 2021a). The anti-tumor properties of SCFAs were also demonstrated on lung cancer cell lines *in vitro*. Kim et al. showed that propionate attenuated lung cancer cell proliferation by partially reversing the balance between two proteins: survivin (anti-apoptotic, overexpressed in cancer cells), and p21 (pro-apoptotic, silenced in cancer cells; Kim et al., 2019). However, in-depth *in vivo* studies are needed to closely relate the effect of propionate to cancer treatment.

The research on the immunomodulatory function of metabolites other than SCFAs in the context of lung disease is scarce. One such example is desaminotyrosine (DAT), a flavonoid degradation product of intestinal bacteria. Steed et al., showed that the prophylactic treatment with DAT in drinking water protected mice against mortality and weight loss caused by influenza virus infection. However, the timing of the metabolite administration appeared to have a critical impact since its application 2 days after the onset of infection worsened mouse survival. Mechanistically, DAT, when given prophylactically, enhanced type 1 IFN signaling and limited tissue damage in a phagocyte-dependent manner (Steed et al., 2017). Another example of a metabolite active in the airways is p-cresol sulfate (PCS), shown to protect mice against allergic airway inflammation. PCS, the end product of L-tyrosine metabolism by the gut microbiota, reached the lungs *via* the bloodstream and acted upon airway epithelial cells to inhibit the production of a dendritic cell chemoattractant, CCL20. Mice treated with PCS intravenously displayed impaired migration of dendritic cells and, in turn, reduced type 2 inflammation (Wypych et al., 2021). Notably, PCS was negatively correlated with asthma in two human birth cohorts, involving 237 and 411 children, respectively (Kelly et al., 2018). Another bacterial metabolite protective in type 2 immunity is D-tryptophan, produced by *L. rhamnosus* GG or *L.casei* W56. Feeding mice with this amino acid ameliorated Th2 responses in the lung and airway hyperreactivity. This effect was linked with the capacity to reverse the inflammation-induced loss of bacterial diversity in the gut as well as the induction of regulatory T cells (Kepert et al., 2017). Finally, a product of L-histidine metabolism, imidazole propionate (ImP), protected mice against radiation-induced cardiopulmonary injuries. ImP administered *via* oral gavage accumulated in the airways and inhibited pyroptosis in lung cells by suppressing the NF-κB-GSDMD signaling pathway. In addition, it increased the expression of F-actin in lung epithelial cells, improving barrier integrity and tissue regeneration (Chen et al., 2021b). An example of the metabolite that might play a detrimental role in lung disease (aside from SCFAs discussed earlier) is fumarate, shown to promote biofilm formation of methicillin-resistant *S. auerus* clinical isolates (Gabryszewski et al., 2019). Mice infected with *S. aureus* strains incapable of fumarate formation had attenuated inflammatory response (the production of IL-6, TNF-*α*, or IL-1*β*) and a reduced bacterial burden (Gabryszewski et al., 2019).

Collectively, the examples described above point to the immunomodulatory potential of microbial metabolites in the airways (summarized in Table 1). Short-chain fatty acids are most studied in this context, but recent progress unraveled several new metabolites active in the lungs. This holds promise for their development as drugs, which would be of fundamental importance from the clinical point of view, and could constitute an alternative to other microbiome-based approaches.

**Table 1:** Microbial metabolites in mouse models of lung disease.

**Metabolite**	**Metabolite-producing bacterial taxa**	**Mechanisms of action**	**Effect/disease**	**References**
12,13-diHOME	*Enterococcus faecalis, Bifidobacterium bifidum, Streptococcus, Lactobacillus*	(1) Modulates DCs *via* PPAR-γ	Exacerbates allergic airway inflammation	Levan et al., 2019
Acetate	*Bacteroides acidifaciens*	Maintains Treg pool by inhibiting histone deacetylase nine activity and stabilizing Foxp3 expression.	Ameliorates allergic airway inflammation	Thorburn et al., 2015
Acetate	–	Enhances growth of *P.aeruginosa*	Might contribute to rhinosinusitis	Cho et al., 2020
Butyrate	*Bacteroidetes phylum*	(1) Reduces lung pathology by limiting neutrophil recruitment (2) Boosts CD8^+^ T cell anti-viral responses (effect mediated by FFAR3-dependent changes in CD8^+^ T cell metabolism)	Increases survival of influenza virus-infected mice.	Trompette et al., 2018
Butyrate or cocktail consisting of butyrate, acetate and propionate	*Clostridiaceae, Lachnospiraceae, and Ruminococcaceae*	(1) Inhibits the production of IL-4 from CD4^+^ T cells (acting directly on CD4^+^ T cells) (2) Induces anti-inflammatory gene expression profile in DCs, resulting in attenuated DC activation and migration	Reduces allergic airway inflammation	Cait et al., 2018
Butyrate, or propionate	–	(1) Regulates iNOS expression in airway epithelial cells in a concentration-dependent manner: -Augments it at 0.5 mM -Suppresses it at 25 mM (2) Regulates *P. aeruginosa* growth (propionate) in a concentration-dependent fashion: -Enhances it at 3.125–12.5 mM -Inhibits it at 50–100 mM	Might modulate lung inflammation in cystic fibrosis	Ghorbani et al., 2015
Butyrate/propionate	*Lachnospiraceae, Streptococcus and Lachnoclostridium*	Enhances recruitment of Th17 cells to the lungs *via* induction of CCL20 expression in lung endothelial cells.	Attenuates lung metastasis of melanoma cells	Chen et al., 2021a
Conjugated linoleic acid	Bifidobacterium/propionibacterium	Sustains expression of PPAR-*γ*	Ameliorates allergic airway inflammation	Jaudszus et al., 2008
Desaminotyrosine	*Clostridium orbiscindens*	(1) Augments type I IFN signaling pathway in phagocytes (2) Reduces lung immunopathology	Increases survival of influenza virus infected mice.	Steed et al., 2017
D-tryptophan	*Lactobacillus rhamnosus* GG*Lactobacillus casei* W56	1) Reverses inflammation-induced loss of bacterial diversity2) Increases the pool of Treg cells	Ameliorates allergic airway inflammation	Kepert et al., 2017
Fumarate	*Staphylococcus aureus*	Enhances biofilm formation	Promotes *S. aureus* pneumonia	Gabryszewski et al., 2019
Imidazole propionate	*Akkermansia muciniphila, Lactobacillus reuteri, Clostridium* sp. *Cuiture-41, lachnospiraceae bacrerium 615, Ileibacterium valens, Helicobacter bills*	(1) Inhibits pyroptosis in lung cells *via* inhibiting inflammasome activation (2) Enhances the expression of F-actin in lung epithelial cells, improving barrier integrity	Protects against cardipulmonary injury caused by radiation	Chen et al., 2021b
Propionate	Taxa belonging to *Bacteroidetes phylum*	Enhances differentiation of DCs with an impaired ability to activate lung T_H_2 effector cells	Ameliorates allergic airway inflammation	Trompette et al., 2014
Propionate	–	Induces apoptosis and cell cycle arrest by regulating expression of survivin (decreased) and p21 (increased)	Suppresses proliferation of lung cancer cell lines	Kim et al., 2019
P-cresol sulfate	*Prevotella* MGM1	Inhibits the production of CCL20 *via* uncoupling TLR4 and EGFR cross-talk in airway epithelial cells	Ameliorates allergic airway inflammation	Wypych et al., 2021
				
Sphingosine-1-phosphate	–	Increases expression of Th2/17 cytokines in the lungs	Increases airway hyperresponsiveness	Roviezzo et al., 2010
Ursodeoxycholic acid	–	Skews T helper cell pool towards Th1 subset	Ameliorates allergic airway inflammation	Willart et al., 2012

## Therapeutic Advantages and Disadvantages of Postbiotics

Unraveling the importance of the microbiome in shaping health and disease reinforced the old and sparked new ideas to improve human health through microbes. First, Elie Metchnikoff’s postulates from the beginning of the 20th century about improving health with certain foods and bacteria suddenly reemerged. As proposed by Metchnikoff, consumption of yogurt and soured milk, or administration of lactic acid-producing bacteria to bring health benefits gained a tremendous amount of attention (Mackowiak, 2013; Rokana et al., 2017; Mowat, 2021). Soon after the fermented milk hypotheses, other diet hypotheses emerged. Linking the rural environment with the protection from asthma (“the farm effect”) led to the identification of unpasteurized milk consumption as a key component associated with this protection (van Neerven et al., 2012; Wypych and Marsland, 2017). The discovery of anti-inflammatory properties of dietary fibers, and their microbiome-dependent mode of action, resurrected the medicinal use of fiber supplements to improve human health (Sawicki et al., 2017). Though backed up by observational studies and animal models, trials assessing the therapeutic value of the aforementioned dietary interventions in the disease context are either lacking or scarce.

For example, no clinical trials assessing the efficacy of unpasteurized milk interventions in the context of respiratory disease have been published so far (as of January 2022, according to https://trialsearch.who.int/). In the case of soluble fiber intervention, promising preliminary data were collected. A combination of inulin and probiotic yogurt improved lung function and reduced the levels of inflammatory biomarkers (exhaled nitric oxide, sputum IL-8 concentration, sputum neutrophil, lymphocyte, and macrophage counts) in adults with asthma 4 h after the intervention (Halnes et al., 2017). In a follow-up study, a probiotic-free formulation of inulin was used, allowing to dissect the effect of dietary fiber from the effect of probiotics. Again, an improvement of asthma outcomes was observed (based on patients’ questionnaires and frequency of eosinophils in the sputum) after 7 days of treatment (McLoughlin et al., 2019). Though certainly promising, these studies were performed on a very small scale, with only 29, or 17 subjects, respectively. This calls for launching a large, multi-center clinical trial that would support the observed trends.

Finally, the clinical efficacy of the soured milk and currently approved probiotics interventions have been rather disappointing. In general, they pointed to low or very low quality of the evidence that would support the beneficial effect of probiotics in lung disease (Berthon and Wood, 2015; Hao et al., 2015; Suez et al., 2019; Wypych et al., 2019). Also, substantial variability among participants was noted (Hao et al., 2015). It is not entirely clear why probiotics might confer beneficial effects in some people but not in others. One of the reasons might be that probiotics, as live organisms, adapt their function in response to new local environments (different in each person in terms of the microbiota composition and immune-metabolic state). Thus, the “probiotic” function of a certain microbe may be lost, for example, due to undesired interactions with other microbes (Suez et al., 2019). Although direct mechanistic studies to support this possibility in the case of known probiotics are lacking, such a scenario was indeed demonstrated in the case of *Candida albicans*, a fungus that inhabits the mucosal surface of approximately 30% of individuals without causing harm, but that becomes pathogenic under certain circumstances, for example, when the immunity of the host declines (Plantinga et al., 2012). As demonstrated by Tso and colleagues, the virulence of *C. albicans* depends on the intact intestinal microbiota of mice. When the microbiota was disturbed by antibiotic treatment, *C. albicans* not only became avirulent but gained beneficial properties and protected the host against a variety of infections (Tso et al., 2018). Although the key species within the microbiota that maintained the pathogenicity of *C. albicans* remain unidentified, this example underlines the possibility that microbiota constituents might halt the beneficial effect of the administered probiotics in some individuals.

In a similar vein, the resident microbiota might prevent the beneficial effect of probiotics by preventing their colonization within the host. Indeed, many studies pointed to the transient nature of the probiotic existence in the gut (Alander et al., 1999; Goossens et al., 2006; Crittenden et al., 2009; Gianotti et al., 2010; McFarland, 2014; Kristensen et al., 2016; Suez et al., 2019). This is expected in the light of the colonization resistance, whereby intact microbiota prevents the growth of pathogenic microorganisms *via* direct competition for nutrients and space and indirect stimulation of the host’s immunity (Wypych and Marsland, 2018). Although beneficial in the context of opportunistic pathogen invasion, the colonization resistance may come at the price of inhibiting the rationally designed effect of probiotic interventions.

Finally, the extent of probiotics’ effect on extraintestinal tissues relies, at least in part, on the capacity of their products to be absorbed in the blood and distributed to other tissues (Wypych et al., 2019). Thus, bypassing the gut constitutes yet another obstacle to overcome. Indeed, the concentrations of short-chain fatty acids were shown to decline from 131 to 79 mM/kg along the colon–caecum passage to 375–148 μM/l along the portal–hepatic–peripheral blood veins (Cummings et al., 1987). In tissues distal from the gut, the concentrations of bacterial products are expected to decline even further, sometimes even to undetectable levels. For example, a recent study reported a lack of significant concentrations of SCFAs in healthy airways (Yue et al., 2020). Therefore, a potentially immunomodulatory effect of a microbial metabolite in the airways under homeostatic conditions may be hindered by the minute concentrations of the compound at this site.

In the light of the above, applying microbial metabolites locally at the site of interest (e.g., *via* inhalation into the airways) might overcome the problem of the metabolite transit along the gut–lung axis. Therefore, a key feature of metabolites that appears to be pharmacologically attractive is their capacity to bypass the gut, for example, by their local application in a form of a nasal spray. Testing intranasal regimens of metabolite administration in preclinical settings might set the scene for developing microbial metabolites into therapeutics against respiratory diseases. The second feature of metabolites that makes them attractive drug candidates is their ability to trigger specific immunological pathways. This may better ensure their safety, and efficacy in comparison to live therapeutics that trigger multiple (and often undefined) pathways and that may change their function when adapting to a new environment. Finally, the duration of metabolites’ action could be easily estimated by the bioavailability/stability of the administered compound and controlled by the designed dosage regimen. In the case of any adverse effects, the treatment could be aborted, and side effects minimized. This is an important asset not guaranteed by the administration of live microorganisms.

## Conclusion

Taken together, the examples described above illustrate that microbial metabolites constitute an untapped resource of powerful immunomodulators that can be harnessed to treat lung disease. SCFAs, which have dominated the field so far, are clearly among the most promising candidates at the moment, but new immunomodulatory metabolites active in the airways have recently started to be identified. Although they still constitute only the tip of an iceberg of what remains undiscovered, further progress in this field will be fueled by developing better metabolomics tools to interrogate the microbiome, as exemplified by Han and colleagues (Han et al., 2021). Screening for the immunomodulatory properties of unraveled metabolites *in vitro* and in animal models will be inevitable to generate therapeutic leads, and their optimization will open up the possibility to design clinical trials. In the long run, this strategy might translate into the development of microbial metabolites as drugs.

## Author Contributions

EB and TW wrote the manuscript. EB prepared the figure and the table. All authors contributed to the article and approved the submitted version.

## Funding

TW is supported by grants from National Science Centre (SONATA 16, grant no. 2020/39/D/NZ6/02146 and OPUS 21, grant no. 2021/41/B/NZ6/02219).

## Conflict of Interest

The authors declare that the research was conducted in the absence of any commercial or financial relationships that could be construed as a potential conflict of interest.

## Publisher’s Note

All claims expressed in this article are solely those of the authors and do not necessarily represent those of their affiliated organizations, or those of the publisher, the editors and the reviewers. Any product that may be evaluated in this article, or claim that may be made by its manufacturer, is not guaranteed or endorsed by the publisher.
